# The Epidermal Growth Factor Receptor Critically Regulates Endometrial Function during Early Pregnancy

**DOI:** 10.1371/journal.pgen.1004451

**Published:** 2014-06-19

**Authors:** Michael J. Large, Margeaux Wetendorf, Rainer B. Lanz, Sean M. Hartig, Chad J. Creighton, Michael A. Mancini, Ertug Kovanci, Kuo-Fen Lee, David W. Threadgill, John P. Lydon, Jae-Wook Jeong, Francesco J. DeMayo

**Affiliations:** 1 Department of Molecular and Cellular Biology, Baylor College of Medicine, Houston, Texas, United States of America; 2 Dan L. Duncan Cancer Center Division of Biostatistics, Department of Medicine, Baylor College of Medicine, Houston, Texas, United States of America; 3 Department of Obstetrics and Gynecology, Baylor College of Medicine, Houston, Texas, United States of America; 4 Clayton Foundation Laboratories for Peptide Biology, Salk Institute, La Jolla, California, United States of America; 5 Department of Molecular and Cellular Medicine, Texas A&M Health Science Center, College Station, Texas, United States of America; 6 Department of Obstetrics, Gynecology and Reproductive Biology, Michigan State University, Grand Rapids, Michigan, United States of America; Institute of Medical Biology, Singapore

## Abstract

Infertility and adverse gynecological outcomes such as preeclampsia and miscarriage represent significant female reproductive health concerns. The spatiotemporal expression of growth factors indicates that they play an important role in pregnancy. The goal of this study is to define the role of the ERBB family of growth factor receptors in endometrial function. Using conditional ablation in mice and siRNA in primary human endometrial stromal cells, we identified the epidermal growth factor receptor (*Egfr*) to be critical for endometrial function during early pregnancy. While ablation of *Her2* or *Erbb3* led to only a modest reduction in litter size, mice lacking *Egfr* expression are severely subfertile. Pregnancy demise occurred shortly after blastocyst implantation due to defects in decidualization including decreased proliferation, cell survival, differentiation and target gene expression. To place *Egfr* in a genetic regulatory hierarchy, transcriptome analyses was used to compare the gene signatures from mice with conditional ablation of *Egfr*, wingless-related MMTV integration site 4 (*Wnt4*) or boneless morphogenic protein 2 (*Bmp2*); revealing that not only are *Bmp2* and *Wnt4* key downstream effectors of *Egfr*, but they also regulate distinct physiological functions. In primary human endometrial stromal cells, marker gene expression, a novel high content image-based approach and phosphokinase array analysis were used to demonstrate that *EGFR* is a critical regulator of human decidualization. Furthermore, inhibition of EGFR signaling intermediaries *WNK1* and *AKT1S1*, members identified in the kinase array and previously unreported to play a role in the endometrium, also attenuate decidualization. These results demonstrate that EGFR plays an integral role in establishing the cellular context necessary for successful pregnancy via the activation of intricate signaling and transcriptional networks, thereby providing valuable insight into potential therapeutic targets.

## Author Summary

Approximately 10% of reproductive aged women are considered infertile. While great strides have been made in assisted reproductive technologies, overall success rates, especially considering the cost, remain low. Studies indicate that due to its sequential nature, nearly 75% of pregnancy failures are due to defects that occur very early in gestation. Therefore, understanding the physiological changes that occur in the endometrium during this period and how those changes are regulated is of paramount importance if we are to improve our ability to address female reproductive health concerns. We investigated a family of growth factor receptors and identified one that critically regulates the growth and survival of the endometrium in response to the implanting embryo. Furthermore, we used unbiased approaches to identify which signaling pathways and genetic networks are activated downstream of this receptor to execute each of the processes necessary for a successful pregnancy. Understanding the mechanisms and genetic networks with which pregnancy is regulated is a prerequisite to the development of effective pharmaceutical therapeutics.

## Introduction

Human reproduction is remarkably inefficient with a mere 30% of human chorionic gonadotropin (hCG) positive pregnancies resulting in live birth [1]–[3]. Furthermore, an estimated 9% of couples are considered infertile [4]. Pregnancy is a complex, sequential series of events including fertilization, blastocyst attachment and implantation, uterine decidualization, placentation, and parturition (reviewed in [5]). Defects or a failure in any one process results in a ripple effect of detrimental consequences and potentially pregnancy demise [6]. Detailed studies assessing at which stage of pregnancy failures occur indicate that approximately 30% are lost preimplantation, primarily due to genetic defects in the embryo [7]–[9]. Another 30% of pregnancies are lost in the immediate few weeks following implantation [7], and there is a remaining 9–12% risk for women under 35 years of age to miscarry between 6–12 weeks [1]. Moreover, 1–2% of couples, nearly one-third of which experience clinical depression, suffer from 3 or more consecutive pregnancy losses [10]. When women with recurrent pregnancy loss finally do maintain a pregnancy, they are at increased risk for many adverse obstetric outcomes including preterm birth, placenta previa and low birth weight [11]. While some phenomena like embryonic chromosomal abnormalities have been identified, 50–75% of couples with recurrent pregnancy loss show no identified causes [12].

Furthering the understanding of the mechanisms regulating early pregnancy is of paramount importance. The preeminent factors governing female reproductive health are the steroid hormones progesterone and estrogen. While estrogen is known for its mitogenic effects and temporal influence on the window of receptivity [13], progesterone is the predominant hormone of pregnancy (reviewed in [14]). The use of transgenic and knockout animals has done much to advance our understanding of the molecular mediators of pregnancy, including those regulated by progesterone. Particularly compelling was the identification of a signaling axis in which the progesterone receptor (*Pgr*)-mediated induction of indian hedgehog (*Ihh*) leads to the stromal induction of the chicken ovalbumin upstream promoter-transcription factor (*Nr2f2*; COUP-TFII), as mice lacking any of these genes are infertile [15]–[17]. Interestingly, previous works indicate that epidermal growth factor (EGF) signaling is downstream of this signaling axis [16], [18].

The spatiotemporal expression of EGF family members during the implantation period has led to the longstanding interest in the role of growth factor signaling in reproduction [19]–[21]. Of particular interest is the heparin-binding epidermal growth factor-like growth factor (HB-EGF, *Hegf1*), due to its expression in the luminal epithelium surrounding blastocysts at the time of attachment and the effects it has on blastocysts [22], [23]. Furthermore, blastocysts express HB-EGF, and it has been shown to influence uterine decidualization [24]. However, maternal ablation of HB-EGF expression did not render mice infertile, rather these females exhibited a deferred implantation window and reduced litter size. The lack of complete infertility led to the speculation that this phenotype was due to compensation by elevated expression of the ligand amphiregulin (*Areg*) [25]. These results, coupled with the fact that triple knockout females lacking expression of *Areg*, *Egf* and *Tgfa* [26] are fertile, together suggest that the ligands may act in a redundant fashion and led to the hypothesis that the receptors are the rate-limiting factor.

There are four members of the ERBB family of receptor tyrosine kinases: the epidermal growth factor receptor (*Egfr/Erbb1*) and v-erb-b2 erythroblastic leukemia viral oncogene homolog 2–4 (*Her2*, *Erbb3* and *Erbb4*, respectively). Whole animal knockouts of ERBB receptors results in embryonic lethality, demonstrating the critical role in physiology these receptors play [27]–[32]. *Erbb1-3* are expressed in the mouse endometrium while *Erbb4* localizes predominantly to the muscular myometrium and has been shown to not be critical for fertility [33]–[36]. In the present study, we examined the role of *Erbb1-3* in the endometrium using loss-of-function approaches in mice and primary human endometrial stromal cells and determined that that EGFR critically regulates endometrial function.

## Results

### *Egfr* ablation results in severe subfertility

**Figure 1:**
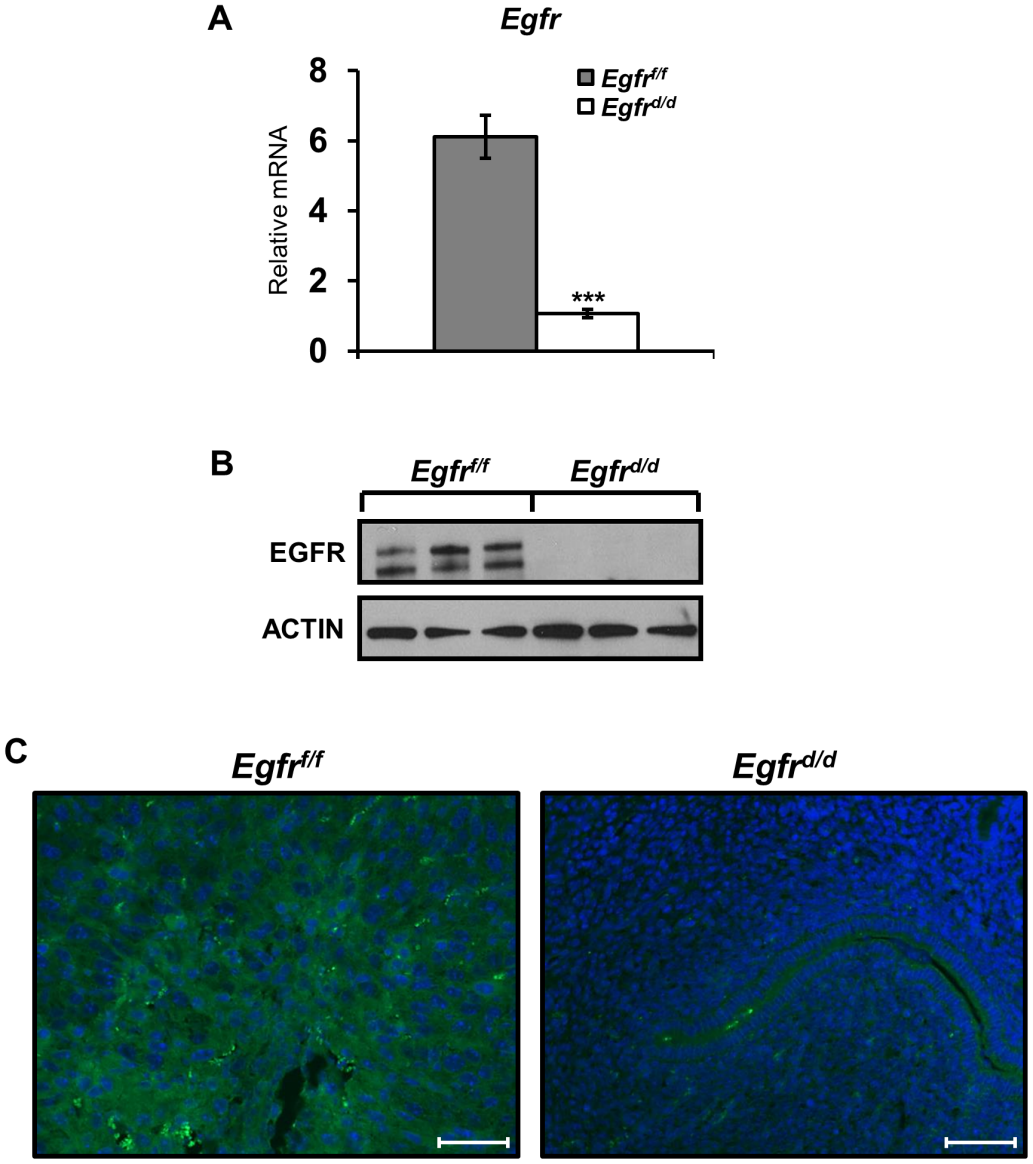
*Egfr* is effectively ablated in the murine uterus. Successful generation of an *Egfr* conditional knockout (*Egfr^d/d^*) using the *Pgr^Cre^* mouse model as determined by (**A**) qPCR; average +/− SEM. ***-p<0.001, (**B**) western blot and (**C**) immunofluorescence. Scale bars 50 µm.

To circumvent the embryonic lethality exhibited by whole body ERBB knockout animals, mice carrying floxed alleles [37]–[39] were crossed with the *Pgr^Cre^* mouse model [40], effectively ablating *Erbb* expression in the uterus (Figure 1). To determine the impact that *Erbb* ablation has on female fertility, control (*Pgr^+/+^Erbb^f/f^*; f/f) and knockout females (*Pgr^Cre/+^Erbb^f/f^*; d/d) were mated with wild type males of proven fertility in a 6-month fertility trial. Uterine ablation of *Egfr* (*Erbb1*) had a major impact on endometrial function, causing infertility in three of seven females tested, while four of the seven females produced only 10 pups over a six month period. In contrast, ablation of either *Her2 (Erbb2)* or *Erbb3* resulted in only a modest impairment in the average litter size (Table 1). Given that of the Erbb receptors expressed in the endometrium, ablation of *Egfr* had the most significant impact on fertility, we chose to focus our investigation on this receptor.

**Table 1:** *Egfr* (Erbb1) is the predominate endometrial Erbb receptor of female fertility.

Genotype	Females	Pups	Litters	Pups per Litter	Litters per Female
***Erbb1^f/f^***	7	342	42	8.24+/−0.34	6.00+/−0.31
***Erbb1^d/d^***	7	10	8	1.50+/−0.50	1.14+/−0.46
***Erbb2^f/f^***	5	186	23	8.12+/−0.48	4.60+/−0.51
***Erbb2^d/d^***	5	128	25	5.15+/−0.48	5.00+/−0.45
***Erbb3^f/f^***	4	181	23	7.91+/−0.50	5.75+/−0.25
***Erbb3^d/d^***	5	137	24	5.79+/−0.40	4.80+/−0.20

6 month breeding trial using wild-type males. Average numbers are +/− the SEM.

### No overt defects in ovarian or oviduct function using the *Pgr^Cre^* to ablate *Egfr* expression

The *Pgr^Cre^* not only excises alleles in the uterus, but is also active in the oviduct and during a brief window in the corpus luteum [40]. Prior to investigating the loss of *Egfr* in the endometrium, the role of *Egfr* in ovarian, luteal and oviductal function was interrogated. To examine the ability to produce oocytes, 3-week old females were stimulated with gonadotropins and mated with wild-type males. At day 0.5 of pregnancy, ova were recovered from the oviduct and no differences in the number present were observed between genotypes (Figure S1A). To assay ovulation, fertilization and blastocyst transport through the oviduct, naturally cycling adult females were mated with wild-type males and blastocysts were flushed from the uterus. Though a trend toward a lower number of uterine blastocysts was observed in *Egfr^d/d^* females, the difference was not statistically significant (Figure S1B). Importantly, following flushing of the uterus, the oviducts were dissected to examine whether any blastocysts failed to be transported to the uterus and none were observed. Luteal function was examined by measuring the serum concentration of progesterone at day 5.5 of pregnancy, and no differences were observed (Figure S1C). Together, these data suggest that ovarian function is largely intact and the subfertility observed in *Egfr^d/d^* females is due to uterine defects.

### *Egfr* ablation does not inhibit the acute response to hormones

**Figure 2:**
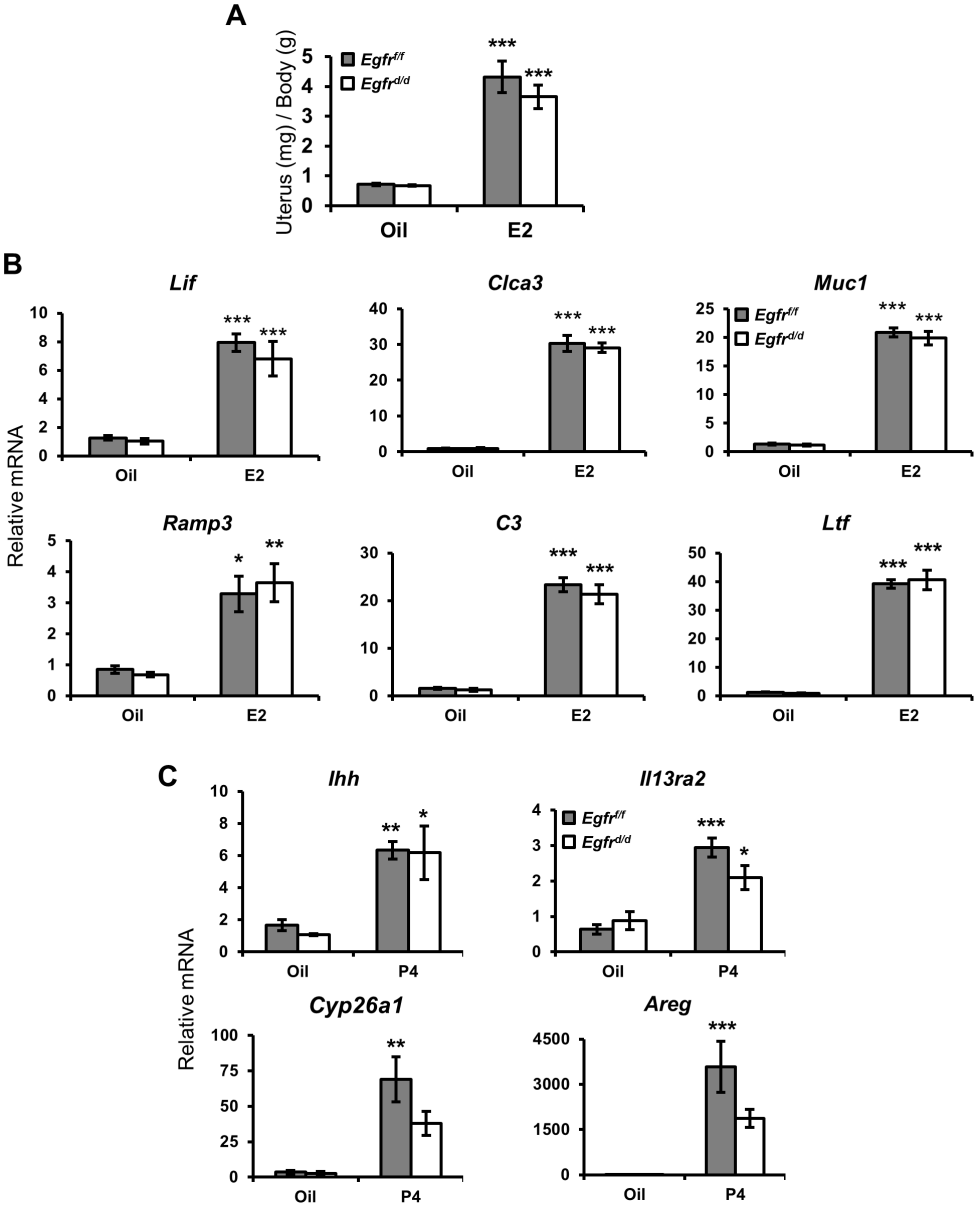
Ablation of *Egfr* does not impair acute hormone responsiveness. Mice were ovariectomized and treated with: (**A,B**) estrogen for 3 d or (**C**) progesterone for 6 h. (**A**) Measurement of the induction of uterine wet weight in response to estrogen. qPCR measurement of hormone target genes after (**B**) 3 d of estrogen or (**C**) 6 h of progesterone. Average +/− SEM. *-*p*<0.05; **-*p*<0.01; ***-*p*<0.001.

We next sought to determine how the loss of *Egfr* impaired female fertility. It is well known that the uterus is a steroid hormone responsive organ, and the actions of estrogen (E2) and progesterone (P4) are absolutely critical for the establishment of a successful pregnancy (reviewed in [14]). Many previous reports have identified a potential link between EGF and E2 action [41], [42]. To determine whether the impairment in fertility observed in *Egfr^d/d^* females was due to deficient E2 action, ovariectomized mice were given three daily injections of E2 and the E2-dependant induction of uterine wet weight and target gene expression were assessed. Surprisingly, there was no difference in uterine weight gain or in E2 target gene expression between *Egfr^f/f^* and *Egfr^d/d^* females (Figure 2A, B). Next, we sought to determine whether the observed subfertility was due to defects in progesterone action. To address this possibility, ovariectomized mice were treated with a single dose of P4 for 6 h and target gene expression was measured by qPCR. As expected, expression of acute P4 target genes *Ihh*, *Il13ra2*, *Cyp26a1* and *Areg* was induced after ligand exposure (Figure 2C). The ability of *Egfr^d/d^* uteri to induce expression of *Ihh*, *Il13ra2*, and *Areg* was comparable to that of controls. The expression of *Cyp26a1* trended toward being reduced, but this difference was not statistically significant.

### *Egfr^d/d^* females exhibit normal expression of preimplantation markers

**Figure 3:**
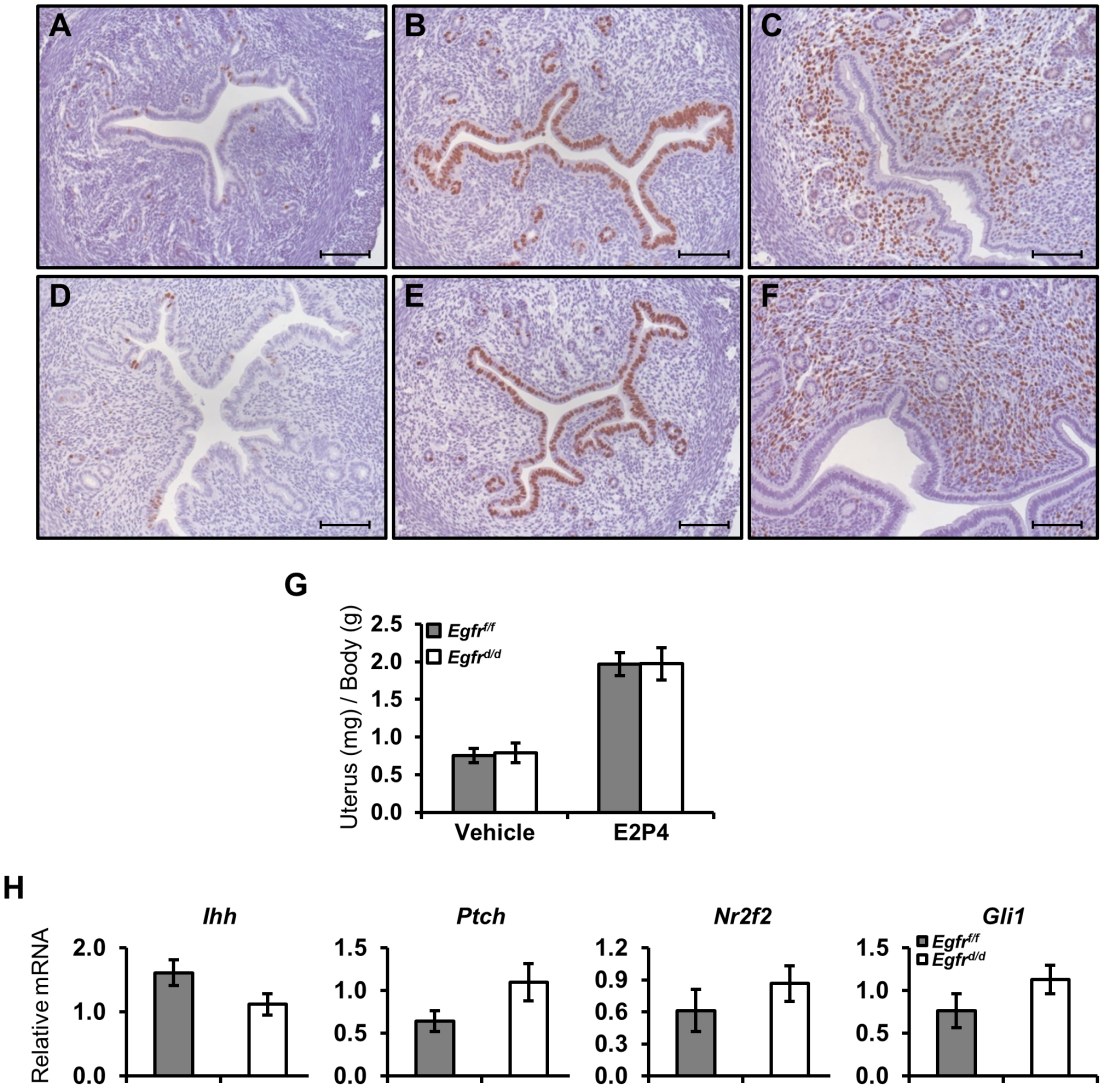
Ablation of *Egfr* does not alter delayed pregnancy proliferation or preimplantation markers. Mice were ovariectomized and treated with exogenous hormones to mimic various stages of preimplantation biology. (**A–F**) Measurement of proliferation by BrdU immunohistochemistry in (**A–C**) *Egfr^f/f^* or (**D–F**) *Egfr^d/d^* mice primed with estrogen and then treated with (**A,D**) vehicle, (**B,E**) estrogen or (**C,F**) estrogen (50 ng) and progesterone for 15 hours. Scale bars: 100 µm. (**G,H**) Mice were treated with 3 days of estrogen (100 ng), given 2 days rest and then treated for 2 days with estrogen (6.7 ng) and progesterone (1 mg) to mimic day 3.5 of pregnancy. (**G**) Measurement of uterine wet weights after oil or hormone treatment. (**H**) qPCR measurement of known preimplantation regulators.

Pregnancy requires the coordinated orchestration of P4 and E2 action, and this first becomes evident in the preimplantation period when the uterus undergoes a switch from predominantly E2-mediated physiology to P4-mediated action. Two days prior to blastocyst attachment, the uterine epithelium is proliferating under the influence of E2. Following ovulation and the luteal production of P4 one day prior to attachment, proliferation ceases in the epithelium while it is induced in the stroma. Exogenous hormones may be given to mice to mimic this period free of ovarian or embryonic influences [43]. With this regimen, *Egfr^f/f^* mice exhibit the characteristic induction of epithelial proliferation with E2 (Figure 3B) as well as the inhibition of epithelial proliferation and concomitant induction of stromal proliferation after P4 treatment as measured by BrdU incorporation (Figure 3C). Under the same hormone paradigm, the response of *Egfr^d/d^* mice did not differ from controls (Figure 3E, F). To further investigate the preimplantation period, and because *Egfr* has been identified to be involved in IHH signaling during the preimplantation window, we also measured the expression level of several genes in this signaling axis. No significant differences in the expression level of *Ihh*, *Ptch*, *Gli1* or *COUP-TFII* were found between *Egfr^f/f^* and *Egfr^d/d^* mice (Figure 3H).

### Early pregnancy failure and implantation site demise is a key factor in female *Egfr^d/d^* subfertility

**Figure 4:**
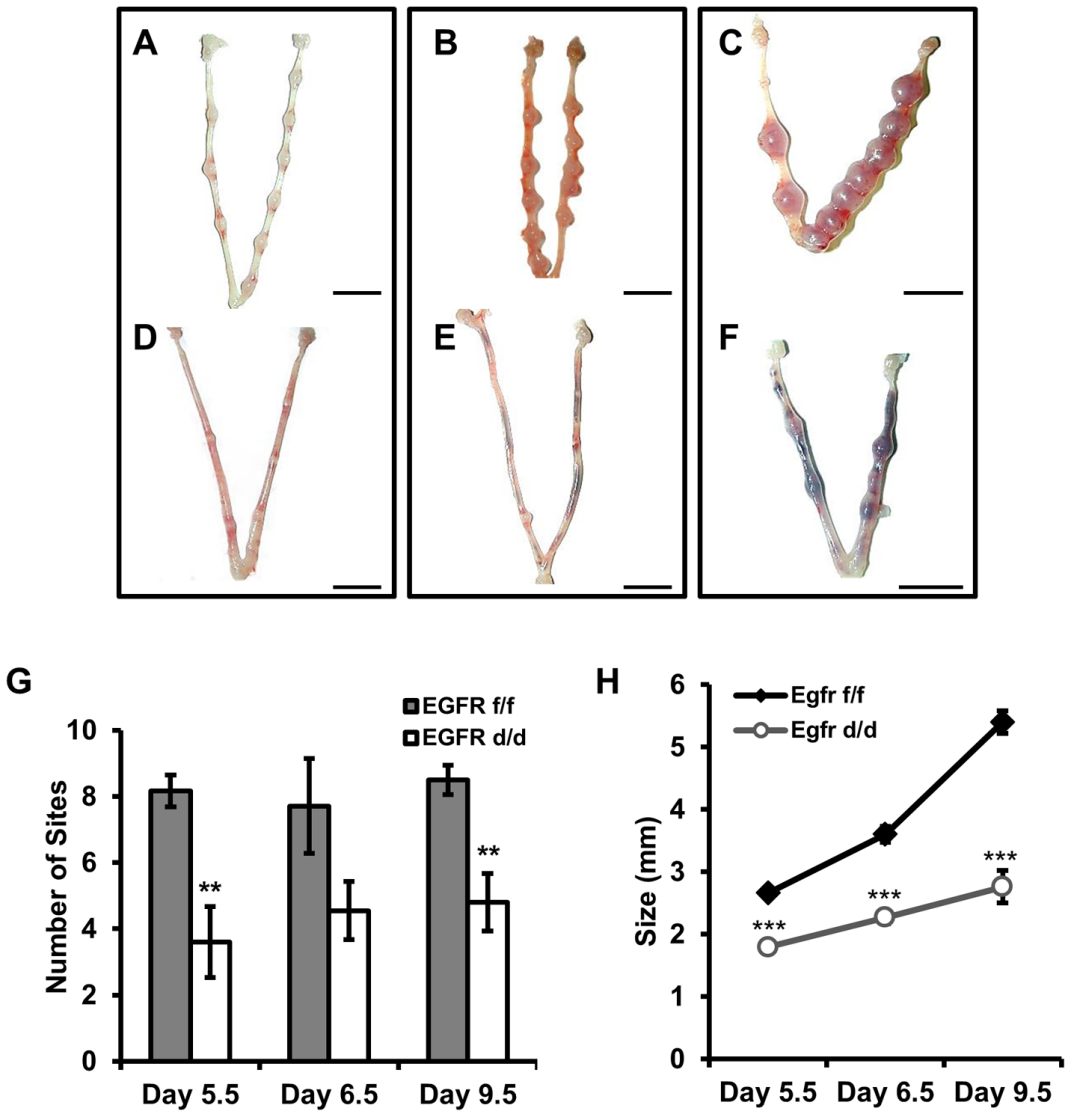
*Egfr* ablation causes blastocyst implantation site demise. (**A–F**) Gross morphology of pregnant uteri. (**A–C**) *Egfr^f/f^* and (**D–F**) *Egfr^d/d^* females were mated with wild-type males. The morning observance of a vaginal plug was considered day 0.5 of pregnancy. Pregnancy was assessed at (**A,D**) d5.5, (**B,E**) d6.5 and (**C,F**) d9.5. Scale bars: 1 cm. (**G**) Average number of implantation sites observed. (**H**) Average lateral diameter of implantation sites. Numbers represent average +/− the SEM. **-*p*<0.01; ***-*p*<0.001.

With no apparent defects during the preimplantation period, we next chose to investigate a potential role for *Egfr* during blastocyst implantation. To address this, control and conditional knockout mice were mated with wild-type males. The morning a vaginal plug was observed was considered day 0.5 of pregnancy, and mice were sacrificed at various time points thereafter. Beginning at day 5.5, one day after blastocyst attachment, implantation sites and decidual balls were readily observable in *Egfr^f/f^* females (Figure 4A). Likewise, implantation sites were visible in *Egfr^d/d^* females (Figure 4D), indicating that the uterus is at least permissive and capable of blastocyst attachment and implantation. However, the average number of observed implantation sites (Figure 4G) and the size of those observed was reduced (Figure 4H). We next asked whether the differences observed at day 5.5 were simply due to a delay in the timing of pregnancy, as occurs in the previously mentioned *Hb-egf^d/d^* model, or if the attenuated implantation reaction would have a ripple effect and manifest as a more severe phenotype later in pregnancy. At day 6.5 of pregnancy, obvious increases in implantation site size can be observed in *Egfr^f/f^* mice (Figure 4B). However, implantation sites in *Egfr^d/d^* mice fail to show a significant increase in diameter (Figure 4E). Furthermore, intrauterine hemorrhaging indicative of embryo resorption is readily apparent in *Egfr^d/d^* mice. Continued observation of pregnancy progression to day 9.5 reveals that while the *Egfr^f/f^* implantation sites develop normally (Figure 4C), those in *Egfr^d/d^* females fail to recover from the early impairments in implantation and continue to be reabsorbed (Figure 4F).

### Implantation site demise is due to a failure in the maintenance and progression of decidualization

**Figure 5:**
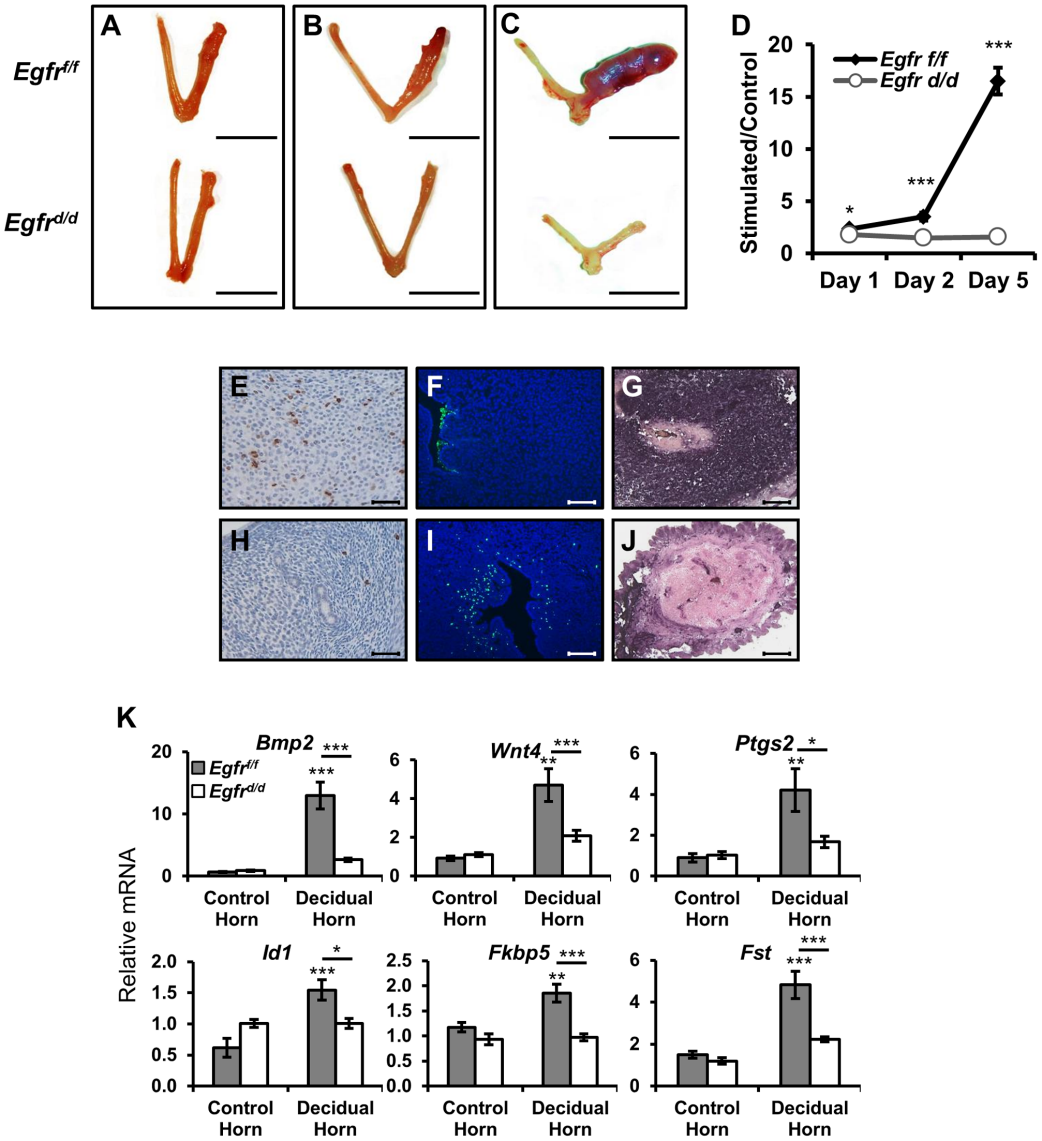
*Egfr* ablation results in major defects in decidualization. Ovariectomized mice were treated with exogenous hormones and a deciduogenic stimulus was administered to one uterine horn. Gross uterine morphology of uteri (**A**) 1 day, (**B**) 2 days, or (**C**) 5 days after deciduogenic stimulus. Scale bar: 1 cm. (**D**) Wet weight ratio of stimulated uterine horn relative to unstimulated horn. Histological analysis of day 2 stimulated uterine cross-sections comparing (**E–G**) *Egfr^f/f^* and (**H–J**) *Egfr^d/d^*. (**E,H**) Proliferative marker phospho-histone H3, (**F,I**) apoptosis via TUNEL assay, and (**G,J**) differentiation via alkaline phosphatase staining. Scale bars: 100 µm (E,H,F,I) and 50 µm (G,J). (**K**) Measurement of the induction of mRNA expression of known decidual regulators on day 1 uteri by quantitative real-time PCR. Average +/− SEM. *-*p*<0.05; **-*p*<0.01; ***-*p*<0.001. n.s. - not significant.

The attachment of the blastocyst to the uterine epithelium and subsequent invasion into the adjacent stroma triggers the proliferation and differentiation of said cells in a process termed decidualization (reviewed in [5]). To investigate the cause of implantation failure, we assessed the ability of *Egfr^d/d^* mice to mount a decidual response following exogenous hormone treatment and mechanical deciduogenic stimulus. One day after stimulation (analogous to day 5.5 of natural pregnancy), the hallmark induction of biomass and increased size of the stimulated horn is apparent in *Egfr^f/f^* mice (Figure 5A). Similar to the findings during implantation, *Egfr^d/d^* uteri do exhibit a response to deciduogenic stimuli, however, one that is dampened. Also similar to implantation, while the control uteri continue to increase in size two days after stimulation (analogous to day 6.5 of natural pregnancy), *Egfr^d/d^* uterine horns fail to progress, and they regress to a point where stimulated horns are indistinguishable from those that were left unstimulated (Figure 5B). When the decidual response five days after stimulation (analogous to day 9.5 of pregnancy) was assayed, the difference between *Egfr^f/f^* and *Egfr^d/d^* mice is greater than 10-fold (Figure 5C,D), which is in contrast to the lack of detectable differences between *Erbb2* or *Erbb3* mice and their respective controls during decidualization (Figure S2).

As mentioned previously, decidualization is a complex process that begins with a wave of proliferation that eventually leads to differentiation. To determine whether defects in any of these cellular functions may be contributing to the decidualization phenotype, we used immunohistochemistry to compare tissue cross-sections of stimulated uterine horns. When day two stimulated sections were stained using an antibody against the mitotic marker phospho-histone H3, positive immunoreactivity was observed in the nuclei of many *Egfr^f/f^* stromal cells (Figure 5E). Strikingly, cross-sections from *Egfr^d/d^* uteri exhibit very few immunopositive cells (Figure 5H). Because it appeared as if though *Egfr^d/d^* mice were able to initiate decidualization but failed to maintain it, we next asked if the observed decrease in proliferation was coupled with increased apoptosis. While the uterine epithelium of sections from control mice were positive for terminal deoxynucleotidyl transferase dUTP nick end labeling (TUNEL), a process known to aid blastocyst invasion, the subepithelial stroma was negative (Figure 5F). Conversely, no staining was observed in the epithelium of *Egfr^d/d^* sections while many of the subepithelial stromal cells did stain positively (Figure 5I). Differentiation of decidualizing cells follows the wave of proliferation. To determine whether defects in proliferation prevented advancement of decidualization to differentiation or if the cells that were able to initiate a response were then able to successfully differentiate, alkaline phosphatase staining was performed. Robust, pervasive dark purple staining is detected in the stroma of decidualizing control mice (Figure 5G), which is largely absent in the knockout sections (Figure 5J).

Previous work has identified important molecular mediators and markers of decidualization, including *Bmp2* [44], [45], *Wnt4* [46], and their effectors. The expression of these genes was measured in both unstimulated and stimulated uterine horns of *Egfr^f/f^* and *Egfr^d/d^* mice. As expected, the expression of *Bmp2*, *Wnt4*, *Ptgs2*, *Id1*, *Fkbp5* and *Fst* show robust induction in the stimulated uterine horn of *Egfr^f/f^* mice. When examining the expression of these genes in *Egfr^d/d^* mice, no significant differences were found between stimulated and unstimulated horns (Figure 5K). Together, these results demonstrate that pregnancy demise in the absence of *Egfr* is due to multiple failures during decidualization.

### Microarray analysis reveals that BMP2 and WNT4 are major mediators of EGFR action

**Figure 6:**
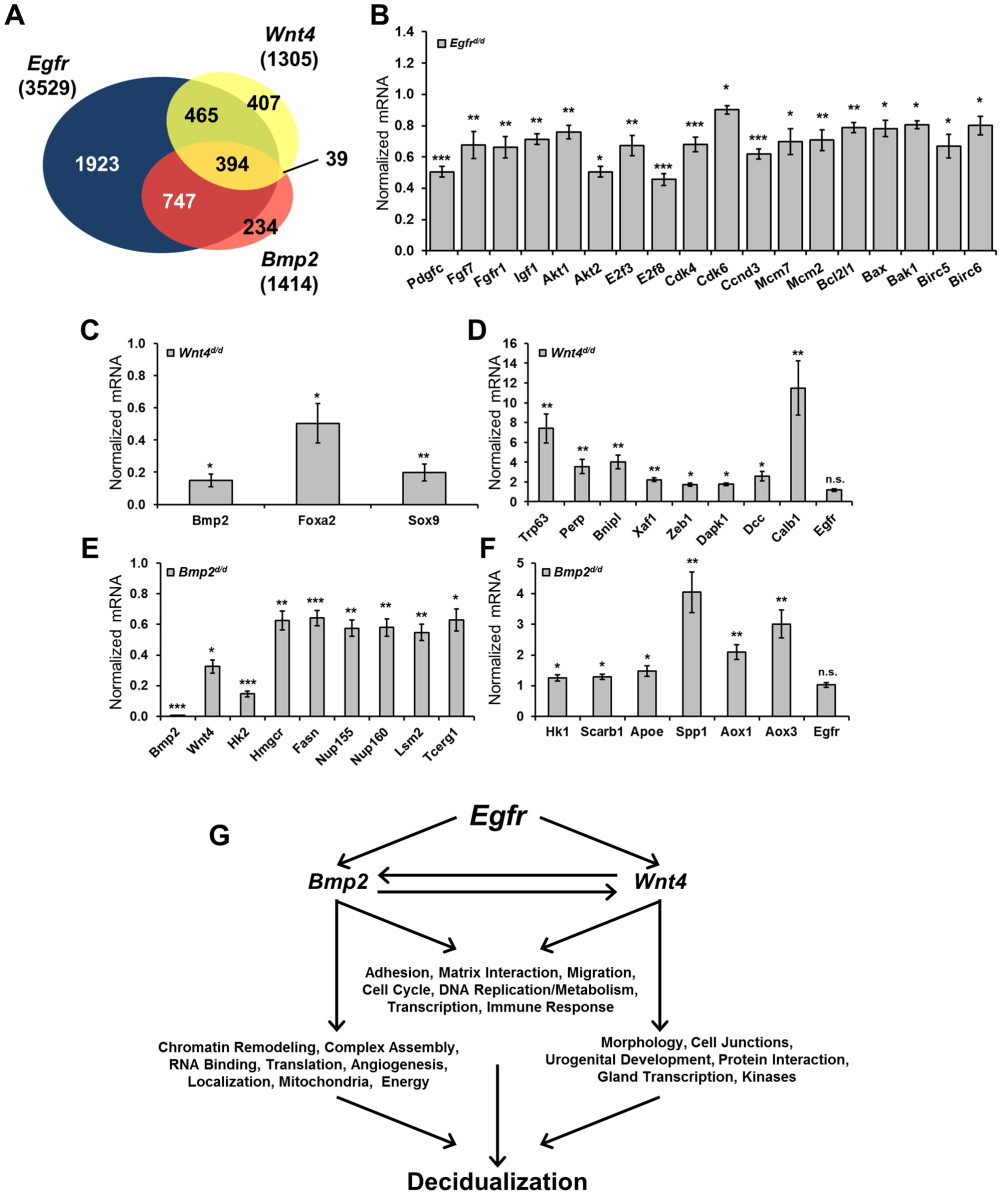
Decidual microarrays reveal BMP2 and WNT as EGFR mediators. (**A**) Venn diagram comparing gene expression signatures from *Egfr*, *Bmp2* and *Wnt4* conditional knockout day 1 decidual uteri. (**B–F**) Quantitative real-time PCR validation of microarray results using independent samples, normalized to control expression. (**B**) Validation of genes altered in the absence of *Egfr*. (**C**) Validation of genes with reduced expression and (**D**) elevated expression in *Wnt4* uteri. (**E**) Validation of genes with reduced expression and (**F**) elevated expression in the absence of *Bmp2*. (**G**) Model depicting which cellular processes and gene ontologies are deregulated in the respective mouse models as determined by DAVID analysis.

To gain further insight into the concatenation of these genes and which cellular processes are affected in their respective absence, microarray analysis was conducted comparing gene signatures between each knockout and their respective controls (*Egfr^d/d^* vs *Egfr^f/f^*; *Wnt4^d/d^* vs *Wnt4^f/f^*) using day 1 decidual uteri. Additionally, published microarray data comparing *Bmp2^d/d^* uteri to controls under identical conditions was used for comparison [45]. Using uniform gene calling (see methods), a total of 3,529 genes were altered in the absence of *Egfr*, 1,414 genes in the absence of *Bmp2* and 1,305 genes in the absence of *Wnt4* (Table 2). More significantly, when the gene signatures of each respective model were compared, significant overlap was observed, with 80.7% and 65.8% of genes regulated by *Bmp2* and *Wnt4*, respectively, also being regulated by *Egfr*. However, a large number of genes (54.5%) regulated by *Egfr* are unique and not altered in the absence of either *Bmp2* or *Wnt4* (Figure 6A). Together, these data implicate a hierarchy in which EGFR signaling acts genetically upstream, while BMP2 and WNT4 act as downstream mediators.

**Table 2:** Summary of microarray gene expression changes.

Genotype	Up	Down	Total
***Egfr***	1980	1549	3529
***Bmp2***	664	750	1414
***Wnt4***	775	530	1305

RNA was isolated from decidual day 1 uteri of *PR^cre^* conditional knockouts and their respective Cre-negative controls. Probes with a fold change ≥+/− 1.4 and a p-value<0.01 were considered significant.

We next used the Database for Annotation, Visualization and Integrated Discovery (DAVID) to determine which gene ontologies and cellular functions were most affected in each model [47], [48]. Analysis on the EGFR gene signature revealed a strong enrichment in cell division, chromatin modification and kinase signaling. Validation of the microarray results was achieved by qPCR of representative genes using independent samples (Figure 6B). Of the 1,305 genes significantly altered in the absence of *Wnt4*, there was a strong enrichment in developmental processes, chondrogenesis and tube development. Genes that were upregulated (Figure 6C) as well as those with reduced induction (Figure 6D) were validated by qPCR. Interestingly, when the genes in common between the EGFR and WNT4 signatures were analyzed, enrichments in morphogenesis, cell junctions, urogenital development and ion channels were found. Although analysis on the *Bmp2* array has been previously published [45], the fact that *Bmp2* was one of the most misregulated genes in the absence of *Egfr* and the gene signatures have extensive overlap led us to investigate which processes were regulated by both genes. Enrichments in chromatin remodeling, metabolism, nucleic acid processing and molecular localization were found, and candidate genes from these processes were validated by qPCR using samples from *Bmp2^d/d^* uteri (Figure 6E, F). When functional analysis was conducted on the 394 genes that were altered in all three microarrays, enrichments for adhesion and matrix interaction, cell cycle, DNA replication and metabolism were found. Importantly, while *Bmp2* and *Wnt4* were misregulated in the absence of *Egfr* (and each in its reciprocal array), *Egfr* is not altered in their absence (Summarized in Figure 6G).

### EGFR regulates human endometrial stromal cell decidualization

**Figure 7:**
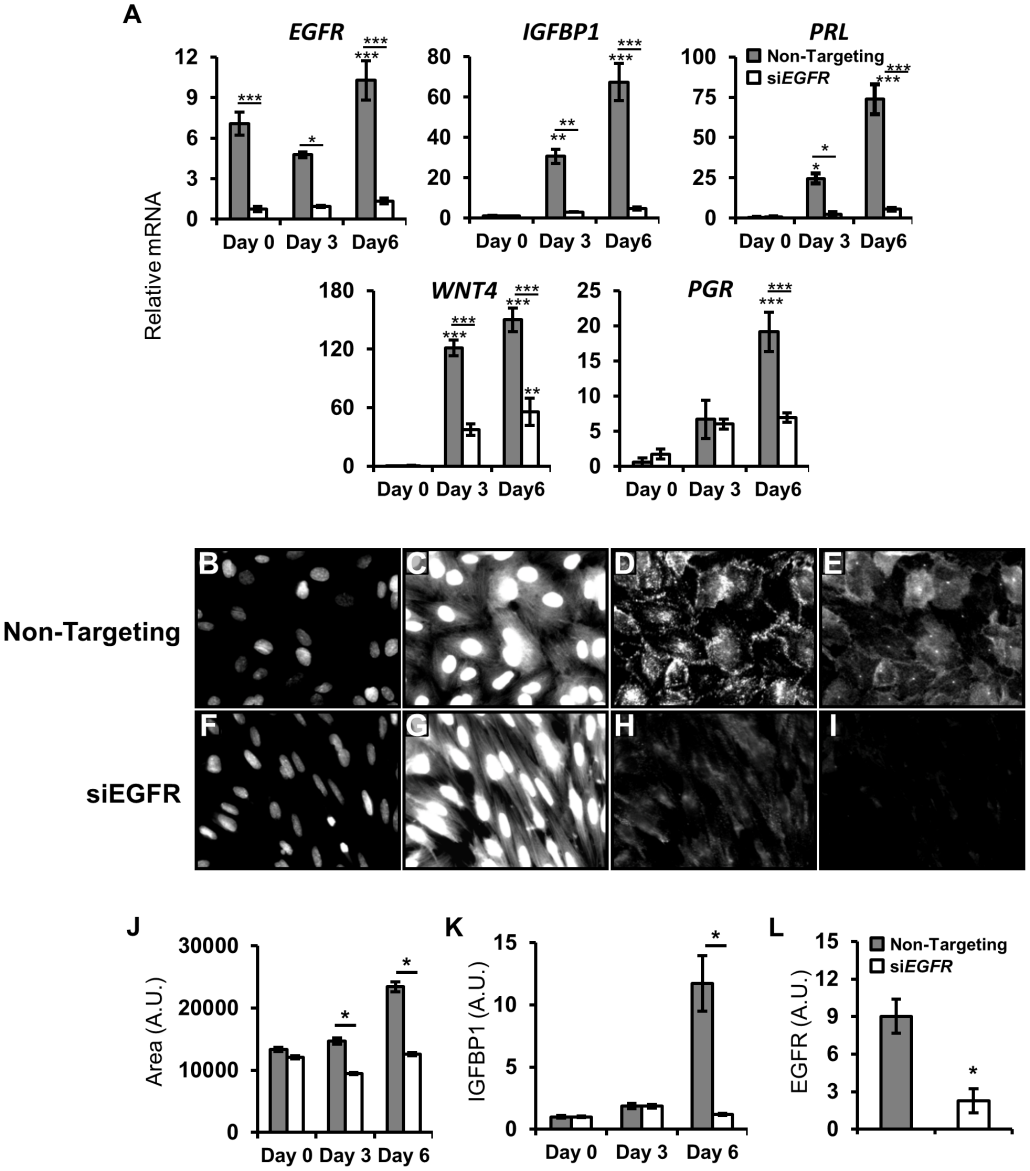
*EGFR* attenuation inhibits human endometrial stromal cell (HESC) decidualization. HESC were transfected with non-targeting or *EGFR* siRNA. After 48 h, HESC were treated with deciduogenic hormones and assessed at day 0, day 3 and day 6. (**A**) qPCR analysis of known decidual markers and regulators. (**B–L**) Day 6 automated quantitative image-based analysis of the effects of siEGFR on decidualization. (**B–I**) Immunofluorescent images of HESC transfected with (**B–E**) non-targeting siRNA or (**F–I**) *EGFR* siRNA after 6 days of deciduogenic hormones. (**B,F**) DAPI staining of nuclei (470 nm, low exposure). (**C,G**) Staining of cell surfaces with CellMask Blue stain (470 nm, high exposure). (**D,H**) Immunolableing of decidual marker IGFBP1. (**E,I**) EGFR immunolabeling. (**J–L**) Quantification of siEGFR and decidual changes. (**J**) Measurement of cell size. (**K**) Induction of IGFBP1 expression. (**L**) Day 6 expression levels of EGFR. Average +/− SEM. *-*p*<0.05; **-*p*<0.01; ***-*p*<0.001.

Primary human endometrial stromal cells (HESC) can be isolated, cultured *in vitro* and treated with a hormone cocktail of estrogen, progestin and cAMP (EPC) to induce decidualization [49]. To test whether *EGFR* is important for human decidualization, we combined this system with a siRNA loss of function approach. HESC transfected with a non-targeting pool of control siRNA and treated with EPC show robust induction of classic decidual markers *IGFBP1* and *PRL*. Conversely, in cells transfected with *EGFR* siRNA, the induction of these markers is markedly impaired (Figure 7A). In addition to changes in gene expression, it is known that stromal fibroblasts undergo a transformation to an epithelioid, cobblestone morphology (reviewed in [50]). While a single representative image can sometimes effectively illustrate the difference between two groups of cells, they are often subjective. To address this, changes in cell morphology were quantified using a high-content, automated imaging system. Cells were transfected with siRNA, treated with EPC and then fixed at various times. Whole cells and nuclear fractions were imaged using CellMask Blue and DAPI, respectively, while antibodies recognizing IGFBP1 and EGFR were used for immunofluorescence. In cells transfected with non-targeting siRNA, large nuclei (Figure 7B), a rounded, cobblestone cell morphology (Figure 7C) and high levels of IGFBP1 (Figure 7D) and EGFR (Figure 7E) are observed. Strikingly, when EGFR expression is significantly attenuated (Figure 7I, L), cells maintain a spindle-like, fibroblast morphology (Figure 7G) and exhibit only basal levels of IGFBP1 expression (Figure 7H). Following segmentation and quantification (see methods), significant increases in cell size (Figure 7J) and IGFBP1 expression (Figure 7K) can be quantified over time, while no significant differences were observed between treated and untreated HESCs lacking EGFR.

### Direct and decidual-dependent kinome alterations following EGFR attenuation in HESC

**Figure 8:**
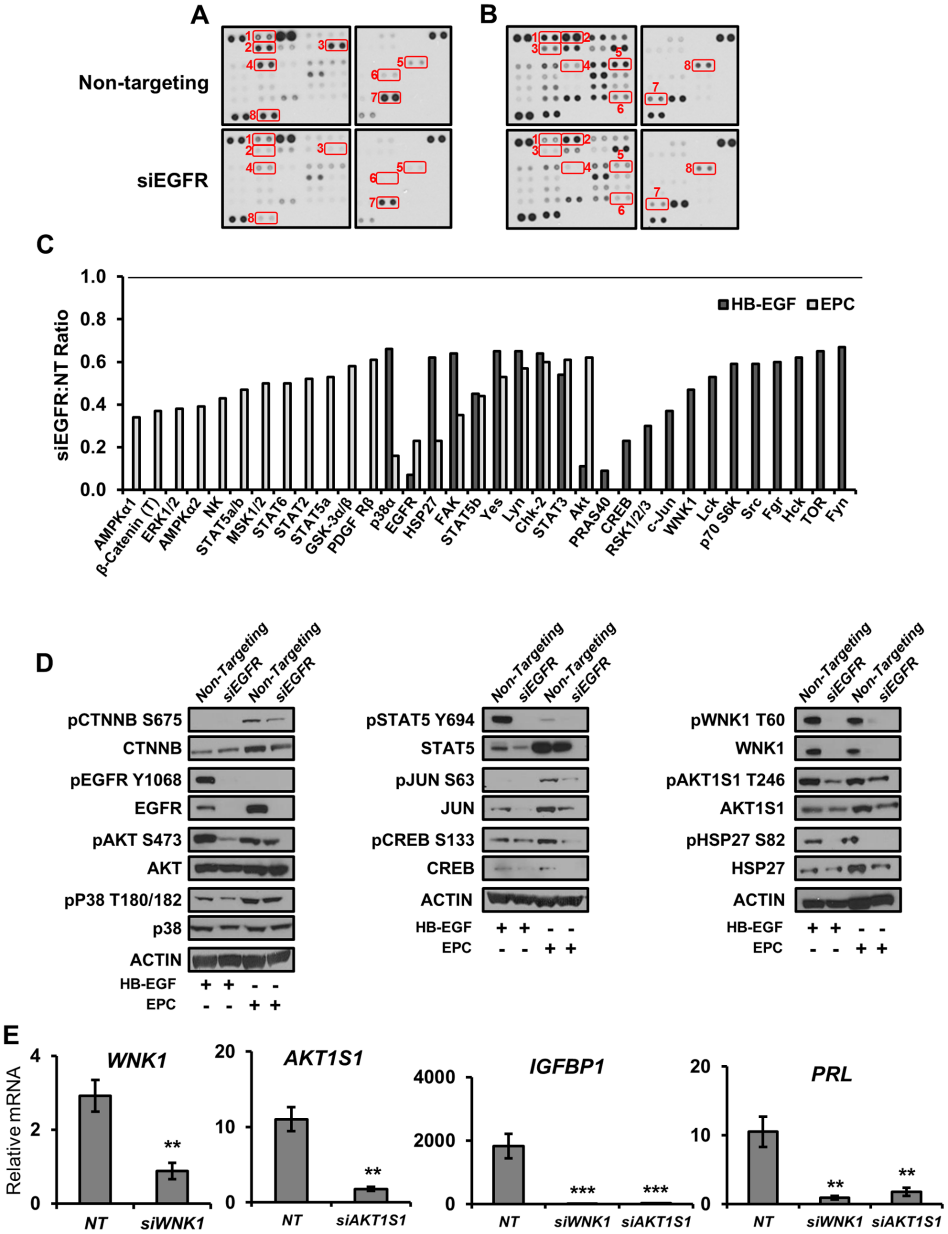
Direct and decidual-dependent kinome alterations following EGFR attenuation in HESC by kinase antibody array. (**A–C**) HESC were transfected with non-targeting or *EGFR* siRNA and treated with (**A**) HB-EGF for 30 mins or (**B**) deciduogenic hormones for 72 h to determine the effect on 44 different kinase substrates via kinase antibody arrays. (**C**) Relative (si*EGFR* vs non-targeting siRNA) quantification of antibodies exhibiting an absolute fold change >1.5. (T) indicates an antibody recognizing total proteins; all others are phosphorylation specific. (**D**) Validation of kinase array results by western blot using independent samples. (**E**) HESC were transfected with non-targeting (NT), *WNK1*, or *AKT1S1* siRNA, treated with deciduogenic hormones for 6 d and the expression of decidual markers was measured by qPCR. Numbers represent average +/− the SEM. **-p<0.01; ***-p<0.001.

The receptor tyrosine kinase EGFR is capable of activating a plethora of signaling cascades, and thus it is likely that the mechanisms by which the aforementioned impairments in decidualization and pregnancy are affected involve phosphorylation. To investigate which phosphorylation events may be altered in the absence of EGFR, we utilized phospho-kinase antibody arrays. HESC were treated with either HB-EGF for 30 minutes (Figure 8A) or EPC for 3 days (Figure 8B) to examine the role of EGFR signaling both acutely and throughout decidualization. Widespread alterations in many phosphorylation events were evident in cells with attenuated EGFR expression. Of the 44 different substrates in the array (Table S1), 11 were altered (absolute fold >1.5) only following HB-EGF treatment, 12 were altered only following EPC treatment, and 11 were altered after both HB-EGF and EPC treatment (Figure 8A–C).

To corroborate the results of the kinase arrays as well investigate whether the absence of EGFR was affecting the expression or phosphorylation of the identified kinases, western blots with independent samples were performed (Figure 8D). Utilizing this approach, we were able to not only confirm that there are widespread alterations in the total amount of phosphorylated kinases present in cellular extracts, but we also identified changes in the expression of several proteins that was dependent on either treatment type or EGFR status. For example, intuitively, the observed decrease in pEGFR is predominantly due to the siRNA-induced depletion of total protein, but it was also observed that in comparison to HB-EGF treatment, EGFR expression is induced 2.5-fold during decidualization. Another example is cJUN, which is also induced during decidualization. Although phosphorylation of cJUN is not detected in samples treated with HB-EGF, total protein expression is virtually lost in the absence of EGFR. During decidualization, total cJUN is reduced by approximately 50% and the protein that remains is phosphorylated at approximately 50% the rate observed in cells with EGFR expression, culminating in an ultimate 75% reduction in phosphorylated cJUN (Figure 8D). Importantly, β-catenin, a downstream effector of WNT signaling, follows a similar pattern; it is induced during decidualization, though to a lesser extent with hypophosphorylation in the absence of EGFR. Additionally, we investigated the function of two members identified in the array that have not yet been reported in the endometrium, *WNK1* and *AKT1S1* (PRAS40), due to their role as signaling intermediaries. Interestingly, the reduction in expression of either member by siRNA resulted in a significant attenuation in the expression of decidual markers *IGFBP1* and *PRL* (Figure 8E). These results identify and provide valuable insight into the complex signaling networks important to endometrial function.

## Discussion

### Erbb signaling, predominantly via EGFR, plays a vital role in early pregnancy

In this report, we hypothesized that the receptors may be the rate limiting factor of Erbb signaling in the regulation of female fertility. We show that EGFR, the only Erbb receptor expressed in the endometrium possessing both functional ligand binding and active kinase activity, plays a critical role in fertility. Modest reductions in litter size observed in the absence of uterine *Her2* or *Erbb3* indicates that they may facilitate EGFR signaling as dimerizing partners but do not play a critical role in pregnancy individually. Growth factors bear their namesake due to the ability to promote growth, and evidence of a relationship between they and the mitogenic hormone estrogen has been well documented [27], [51], [52]. Given the demonstrated relationship between EGF signaling and estrogen, one might intuitively predict that the fertility defects observed in *Egfr^d/d^* mice are due to impairments in estrogen action. Surprisingly, neither epithelial proliferation nor estrogen target gene expression was affected. Taken together, these results suggest that although one of the functions of EGFR may be the activation of the estrogen receptor, estrogen action itself does not require the EGF-receptor.

We did, however, observe significant defects in blastocyst implantation. The observation of implantation sites in the *Egfr^d/d^* uterus one day after implantation indicates that the *Egfr* is not necessary for the recognition of and initial response to the blastocyst, and its absence is still permissive of attachment and invasion. Although a reaction is initiated, it is one that is diminutive and abbreviated, and this may explain the reduction in the number of macroscopically observable implantation sites. We observed EGFR expression in the stromal compartment during this period, and the predominant ligand, HB-EGF, is first expressed in the luminal epithelium at the time of attachment but can then be subsequently observed in proliferating stromal cells [22]. Thus it is intuitive that the primary uterine phenotypes would be in stromal cell function at the site of blastocyst invasion. Defects during the periimplantation period are especially clinically relevant as 30% of human pregnancies are lost during the immediate weeks following attachment [7]. Pregnancy demise due to defects during implantation is a phenomenon that is recapitulated herein when the small implantation sites in *Egfr^d/d^* uteri fail to progress and intrauterine hemorrhaging and fetal resorption ensue.

### Egfr plays a pivotal role in the amplification of decidualization

The decidua plays a critical role in regulating trophoblast invasion, modulating the local immune response, protection from reactive oxygen species and development of the placenta. Consequently, defects in decidualization ripple throughout pregnancy, manifesting in adverse obstetric outcomes such as those in placental position (placenta accreta, placenta previa) and sufficiency (preeclampsia and intrauterine growth restriction), miscarriage, recurrent pregnancy loss, and infertility (reviewed in [6]). Examining the cause of implantation site demise in *Egfr^d/d^* uteri, we observed that EGFR plays a critical role in the amplification and maintenance of decidualization. In results complementing natural pregnancy, an initial response to deciduogenic stimuli did occur, albeit to a lesser extent. However, as the decidua continued to expand in control mice two days after stimulation, proliferation was nearly absent in the stimulated horns of knockout uteri, which had actually begun to regress due to increased apoptosis.

To investigate the global impact on gene expression caused by the loss of *Egfr*, we conducted microarrays one day after deciduogenic stimuli and identified over 3,000 misregulated genes in the absence of *Egfr*. Bioinformatics analysis revealed widespread alterations including genes involved in: growth factor and kinase signaling (*Pdgfc*, *Fgf7*, *Fgrfr1*, *Igf1*, *Akt1*, *Akt2*), the cell cycle (*E2f3*, *E2f8*, *Cdk4*, *Cdk6*, *Ccnd3*), DNA replication (*Mcm2*, *Mcm7*) and cell survival (*Bcl2l1*, *Bax*, *Bak1*, *Birc5*, *Birc6*). The gene networks identified here are in accordance with the phenotype observed where deficient growth signals prevent the advancement of the cell cycle and DNA replication, resulting in a concomitant loss in cell survival. Additionally, we observed significant alterations to two factors previously reported to play a key role in decidualization, *Bmp2* and *Wnt4*.

Of the many BMP ligands and receptors, *Bmp2*, *Bmpr2* and *Acvr1* have been previously shown to play important roles in endometrial function, with both early implantation (*Bmp2* and *Acvr1*) and mid-gestational (*Bmpr2*) defects, some reminiscent of human intrauterine growth restriction [45], [53], [54]. Of the many members of the WNT signaling family expressed during implantation [55], several (*Wnt4*, *Wnt6*, *Sfrp4*, *Dact1*) were found to be misregulated in the absence of *Bmp2* [45]. *Wnt4* is the gene that is most abundantly expressed in the decidua, and its ablation resulted in subfertility due to decreased stromal cell survival and differentiation. Interestingly, the induction of *Bmp2* during decidualization was markedly impaired in the absence of *Wnt4*, leading the authors to propose that *Bmp2* and *Wnt4* function in a mutually activating relationship [46]. Together, these results suggest that the three genes may regulate decidualization in a complex network. To elucidate this relationship, we conducted a microarray on *Wnt4^d/d^* day 1 decidua and compared it to both our *Egfr* microarray and a published *Bmp2* decidual array.

Gene ontology and functional clustering revealed strong enrichments in developmental processes in the 1,305 genes that were altered in the absence of *Wnt4*. These results were fitting since WNT signaling plays a well-documented role in uterine development and differentiation [46], [56]–[59]. Interestingly, when we investigated the relationship between *Egfr*, *Wnt4* and *Bmp2* by comparing all three microarrays, *Egfr* appeared to be the predominant regulator, not only encompassing 80.7% of the *Bmp2* gene signature and 65.8% of the *Wnt4* signature, but also affecting an additional 1,923 unique genes. Furthermore, while *Bmp2* and *Wnt4* were altered in all three models, *Egfr* was not affected in the absence of either *Bmp2* or *Wnt4*, further suggesting a hierarchy in which *Bmp2* and *Wnt4* are mediators of *Egfr* action.

We were also able to identify distinct differences between the enriched functions within the *Egfr-Bmp2* and *Egfr-Wnt4* common genes. The 747 genes regulated by *Egfr* and *Bmp2* exhibit enrichment in general cell functions (RNA trafficking [*Nup155*, *Nup160*], RNA processing [*Lsm2*], transcription elongation [*Tcerg1*]), and metabolism (cholesterol and lipids [*Apoe*, *Hmgcr*, *Cyp39a1*, *Fasn*, *Scarb1*], glucose [*Hk1*, *Hk2*] and respiration [*Aox1*, *Aox3*])). The 465 genes regulated by *Egfr* and *Wnt4* display an enrichment in cell fate and differentiation with processes such as morphogenesis and urogenital development (*Foxa2*, *Zeb1*, *Sox9*, *Trp63*), cell interaction (*Perp*, *Trp63*) and cell survival/death (*Dapk1*, *Dcc*, *Xaf1*, *Bnipl*) being enriched. By comparing three different mouse models, this analysis provides valuable insight into how multiple signaling networks converge in the execution of decidualization and thereby the achievement of a successful pregnancy.

### Attenuation of EGFR causes extensive alterations to kinase signaling, preventing HESC decidualization

Treatment of cultured HESCs with a hormone cocktail of estrogen, progestin and cyclic AMP (EPC) induces *in vitro* decidualization, the hallmarks of which are the induction of *IGFBP1*, *PRL* and transformation from a spindle-like fibroblast to an epithelioid, cobblestone-like morphology [60]. Using molecular and novel image-based approaches, we demonstrated that *EGFR* is critical for human decidualization. Interestingly, while basal and early decidual expression of *PGR* was unaffected by si*EGFR*, the amplification of *PGR* mRNA late during decidualization was blunted. The decidual induction of *PGR* makes it challenging to determine whether EGFR signaling regulates *PGR* directly or if its attenuation is due to the lack of decidualization. It has been reported that predominant EGFR ligands during pregnancy, *Hbegf* and *Areg*, as well as *Egfr* itself, are PGR target genes [21], [25], [61]. An attractive conjecture would be that a feed-forward amplification loop exists in which PGR induces EGFR signaling that in turn feeds back to maintain PGR activation. Such a model would serve as an exquisite sensor of progesterone activity, linking the health of the pregnancy to endometrial function.

Enzymes, particularly kinases, are some of the most commonly targeted proteins for therapeutic manipulation (reviewed in [62]). To better understand which signaling pathways EGFR might be acting through in HESC, we examined the activation of acute targets 30 minutes after growth factor treatment as well as those that are regulated during decidualization after 3 days of hormones. Using phosphokinase antibody arrays and western blot confirmation, we identified a wide array of kinome alterations in the absence of EGFR.

Significant impairments in the rapid activation of members of the SRC cascade (SRC, FAK, FYN, HCK, LCK, LYN, YES), known to be important during HESC decidualization [63], were evident in the absence of EGFR. Additionally, AKT/mTOR signaling (AKT, WNK1, PRAS40, MTOR, P70S6K) as well as MAPK and downstream effectors (P38a, RSK1/2/3, HSP27, JUN, CREB) were inhibited following HB-EGF treatment. While some of these same signaling members (AKT, GSK3b, ERK1/2, P38a, PDGFR, FAK, LYN, YES) were also affected following decidualization, pathways showing more dramatic alteration in the hormone milieu include STAT signaling (STAT2, STAT3, STAT5a/b, STAT6), the response to cellular stress (AMPKa1, AMPKa2, HSP27, MSK1/2), and importantly CTNNB, a downstream WNT effector. The interconnected nature and cross talk that exists between most signaling pathways makes the establishment of definitive causal relationships difficult, but it is clear that the absence of EGFR causes widespread havoc on intracellular signaling and thus prevents the feed-forward amplification of decidualization.

Additionally, we demonstrate that inhibition of EGFR signaling intermediaries *AKT1S1* or *WNK1* also impairs HESC decidualization. The identification of a role for *WNK1* in decidualization is particularly intriguing. WNK1 is a member of the WNK subfamily of kinases that is most prominently known to play important roles in the regulation of ion transporters and electrolyte homeostasis. Mutations in *WNK1* were discovered to cause a familial form of hypertension known as Gordon's syndrome, characterized by increased renal salt reabsorption [64]. Additionally, WNK1 has been shown to regulate the epithelial sodium channel ENaC [65], an interesting finding considering endometrial ENaC has recently been shown to be critical for blastocyst implantation and is markedly reduced in women who experience an unsuccessful *in vitro* fertilization cycle [66]. Although we are the first to show that WNK1 is activated downstream of EGFR signaling in endometrial cells, additional evidence of this relationship exists in that WNK1 was shown to be required for the EGF-dependent activation of ERK5 in kidney cells [67]. While the WNK family is most similar to the MEKK family of kinases, they are atypical in that they do not fit in any of the seven major kinase families [68]. Of the 518 different protein kinases identified in the human genome, 428 of them utilize a highly conserved lysine in subdomain II [69]. In contrast, the WNK family bears their namesake (With No Lysine [K]) due to the absence of this important residue [68], [70]. The atypical catalytic domain of WNK proteins not only confers unique substrate binding but also represents an attractive target for the development of specific small molecules. For example, while the acute nature of targeted periimplantation growth factor supplementation in women with a history of pregnancy complications would likely limit the potential for adverse side effects, EGFR is expressed in many tissues and is well known to play oncogenic roles in the brain, lung, breast, pancreas and colon. Development of pharmaceuticals targeting the atypical kinase domain of WNK1 could potentially confer not only target protein specificity but also reduce non-uterine effects.

### Summary

Our present findings demonstrate that the coordinate actions of EGFR are critical for the successful progression of early pregnancy. The serial, progressive nature of pregnancy prescribes that deficiencies in early events generate ripple effects, potentially leading to pregnancy termination [6]. Thus, identification and modulation of members involved during this period is of paramount importance in the improvement of female reproductive health. We show here that EGFR is active during this window, triggering a multitude of signaling and transcriptional events, and thus it establishes the cellular context necessary for the continued progression of pregnancy. Several studies have implicated a potential a role of EGFR signaling in human pregnancy. A maternal polymorphism in the 5′ untranslated region of the EGF locus was associated with intrauterine growth restriction (IUGR) [71]. Similarly, decreased expression and activation of EGFR is associated with low birth weight and IUGR [72]–[74]. Elucidation of the EGFR signaling network also has implications beyond reproductive health as it is overexpressed in approximately 50% of endometrial tumors and is significantly associated with decreased survival in patients with Type II endometrial cancer [75], [76]. Furthermore, expression of negative regulators of EGFR signaling, TOB1 and MIG6, has been found to be reduced in women with endometriosis [77], [78]. Insight gained herein will aid in the development and advancement in our ability to treat women suffering from reproductive dysfunction.

## Methods

### Mice and human specimens

Floxed Erbb mice were crossed with the progesterone receptor cre mouse model to generate conditional knockout models. All mice were maintained under pathogen-free conditions. Animal handling and surgery was performed according to the NIH *Guide for the Care and Use of Laboratory Animals*. All procedures and protocols were approved by the Institutional Animal Care and Use Committee at Baylor College of Medicine. Typical cohorts consisted of 5–10 mice unless specified otherwise.

Endometrial samples were obtained from the proliferative phase of normally cycling women after written informed consent, under an approved protocol by the Institutional Review Board of Baylor College of Medicine. Histologically normal endometrial tissue samples were obtained by biopsy from subjects between 18 and 45 years of age who had no history of uterine disease. Samples were collected at the room temperature and transported to the laboratory in HBSS containing 1% antibiotic-antimycotic (Life Technologies, Carlsbad, CA, USA) on ice and processed for the endometrial stromal cell isolation as described below.

### Fertility study and murine hormone treatments

Conditional knockout and respective control females were paired with wild-type males of proven fertility (1∶1) at approximately 8 weeks of age. Fertility was assessed by monitoring litter size and frequency over a six month period. Stimulation of ovulation was induced in 3-week old female mice (N = 3 f/f, 5 d/d) by administering 5 IU of pregnant mare's serum gonadotropin intraperitoneally (i.p.; VWR Scientific), followed by 5 IU human chorionic gonadotropin (i.p.; Pregnyl, Organon International) 48 hours later. Females were placed with wild-type males and 24 hours later ova were flushed from the oviducts and counted. Natural ovulation, fertilization, and oviduct transport were measured in naturally cycling adult females mated to wild-type males. At day 3.5 of pregnancy, uteri were excised and flushed and the blastocysts recovered were counted. Serum progesterone at day 5.5 of pregnancy was measured following isolation by centrifugation using blood collected at the time of sacrifice and serum separator tubes (BD). The serum was sent to the University of Virginia Center for Research in Reproduction Ligand Assay and Analysis Core for analysis of P4 by radioimmunoassay. Blastocyst implantation was measured by mating 8 week old females with wild-type males. The morning a vaginal plug was observed was considered 0.5 days post coitus (day 0.5 of pregnancy). Mice were sacrificed at day 5.5 (N = 6 f/f, 5 d/d), 6.5 (N = 7 f/f, 4 d/d) or 9.5 (N = 4 f/f, 5 d/d) of pregnancy. Uteri were excised, imaged and implantation site diameter was measured using a vernier caliper. For all experiments involving exogenous hormones, females were ovariectomized between 6–8 weeks of age and given 10–14 days to recover and purge endogenous hormones. Hormones were dissolved in sesame oil and administered via 0.1 ml subcutaneous injection. The effects of short term hormone were measured by either three daily injections of 100 ng estradiol (E2) or a single 1 mg progesterone (P4) injection. Mice were sacrificed 6 h after the final injection; uteri were weighed and flash frozen. For preimplantation gene expression studies, mice (N = 6 f/f, 4 d/d) were given 3 d of 100 ng E2 followed with 2 d of rest and then a combination of 6.7 ng E2 with 1 mg P4 for 2 days. Mice were sacrificed 6 h after the final injection, uteri were weighed and flash frozen. For the investigation of preimplantation proliferation using the delayed pregnancy model [43], all mice were given 2 days of 100 ng E2 followed by 2 days of rest. At this point, mice were divided into three cohorts (N = 4 per genotype per treatment): vehicle (4 days vehicle [oil] injections), E2 (3 days of vehicle, 1 injection of 50 ng E2) or EP (3 days of 1 mg P4, 1 injection of 50 ng E2 and 1 mg P4). BrdU (GE Healthcare) was injected intraperitoneally 13 hours after the final hormone injection and mice were sacrificed 2 hours later. The artificial induction of decidualization was achieved using previously described methods [79]. Briefly, following 3 days of 100 ng E2, 2 days of rest and 3 days of 6.7 ng E2 and 1 mg P4, mice were given a deciduogenic stimulus of either 50 ul intrauterine sesame oil injection or mechanically via a scratch with a burred needle. Mice were then given daily injections of the combined E2 and P4 until the day of sacrifice (1 day, 2 days or 5 days after stimulus), 6 hours after the final injection. Uteri were imaged, each horn weighed and then flash frozen.

### Histological and immunohistochemical staining

At the time of sacrifice, a mid-portion of uterus was fixed in 4% (v/v) PFA overnight followed by thorough washing with 70% ethanol. Tissues were dehydrated in a graded ethanol series and embedded in paraffin. Sections were cut (5 µm) and mounted on silane-coated slides. Tissues were deparaffinized, rehydrated and boiled in citrate buffer for antigen retrieval. Sections were preincubated in 10% normal goat serum (NGS) in PBS (pH 7.5) for 1–3 hours at room temperature. Tissue sections were incubated overnight at 4°C with primary antibodies specific to phospho-histone H3 (Millipore, 1∶2000). After washing with PBS, sections were incubated with biotin-labeled secondary antibody (Vector, 5 µl/ml) for 1 hour. Sections treated for immunohistochemistry were then incubated with VECTASTAIN ABC solution (Vector Labs) for 30 minutes followed by peroxidase substrate (Vector Labs) for signal development. Immunoreactivity was detected using the DAB substrate kit (Vector) and sections were counterstained with hematoxylin. The TUNEL assay was performed using the Roche *in situ* cell death detection kit (Roche Applied Science) according to manufacturer's instructions. Alkaline phosphatase staining was performed as previously described [46]. Briefly, 16 µm sections were incubated with a 100 mM Tris buffer (pH 9.5) containing 5-bromo-4-chloro-3-indolyl phosphate and nitro blue tetrazolium chloride (Roche Applied Science). Nuclear Fast Red (Vector) was used for counterstaining.

### Western blot analysis

Protein was extracted using lysis buffer (50 mM Tris pH 7.4, 50 mM KCl, 6 mM EDTA, 1% NP-40) with PhosSTOP phosphatase and Complete protease inhibitors (Roche). Protein was separated by electrophoresis using NuPAGE 4–12% Bis-Tris Gels in MOPS buffer (Life Technologies). Proteins were transferred to a PVDF membrane (Millipore) in transfer buffer (25 mM Tris, 192 mM glycine, and 20% methanol; Biorad). Membranes were blocked in 5% nonfat dry milk which was dissolved in tris-buffered saline/tween solution (TBS/T; 20 mM Tris, 150 mM NaCl, pH 7.6, and 0.1% tween-20) and then incubated with primary antibodies overnight at 4°C with gentle rocking. The membranes were incubated with secondary antibody for 1 hour, washed with TBS/T and developed with ECL prime reagents (GE Healthcare).

### Quantitative real-time PCR

Total RNA was extracted using Trizol reagent (Life Technologies), according to the manufacturer's directions. One microgram of the RNA was reverse transcribed into cDNA with M-MLV (Life Technologies). Expression levels of mRNA were measured by TaqMan Universal PCR Master Mix (Applied Biosystems) or FastStart Universal SYBR Green Master mix (Roche) using the ABI 7500 and QuantStudio 12K Flex Real-Time PCR Systems, according to the manufacturer's instructions. Commercially validated Taqman real-time probes and primers were purchased from Applied Biosystems. The sequences of primers used in SYBR qPCR are listed in Table S2. Relative mRNA levels of transcript were calculated by the 2−ΔΔC method, normalized to the endogenous reference (18S), and plotted as mean ± SEM.

### Microarray analysis

Total RNA was isolated using RNeasy kits (Qiagen) from day 1 decidual uteri (see above) according to manufacturer's instructions and three groups of two were pooled for each condition. The integrity of all RNA samples were tested with the Bioanalyzer 2100 (Agilent Technologies). Microarrays were performed by the Genomic and RNA Profiling Core of Baylor College of Medicine. Samples were labeled using the standard Affymetrix linear amplification protocol using the 3′ IVT Express Kit. 250 ng of total RNA was reverse transcribed to produce double-stranded cDNA. The cDNA product was used as a template for the vitro transcription reaction, producing biotin-labeled cRNA. The labeled cRNA was tested for integrity on the Agilent Bioanalyzer and quantified using the NanoDrop ND-1000 spectrophotometer. 15.0 µg of the labeled cRNA was fragmented and re-checked for concentration and size. Hybridization cocktails containing Affymetrix spike-in controls and 15.0 ug of each fragmented, labeled cRNA were loaded onto Affymetrix GeneChip Mouse 430 2.0 arrays. The arrays were hybridized for 16 hours at 45°C with rotation at 60 rpm in the Affymetrix GeneChip Hybridization Oven 640. The arrays were washed and stained with a streptavidin, R-phycoerythrin conjugate stain using the Affymetrix GeneChip Fluidics Station 450. Signal amplification was done using biotinylated anti-streptavidin. The stained arrays were scanned on the Affymetrix GeneChip Scanner 3000. The images were analyzed and quality control metrics recorded using Affymetrix Command Console software version 3.0.0. Microarray CEL files were analyzed using dChip (www.dchip.org, PM-MM model, Quantile normalization). Two-side t-test and fold changes were used to define differentially expressed probes. To obtain a non-redundant list of significantly altered genes, multiple probe sets for a given gene (when present) were averaged. Genes with an absolute fold change ≥1.4 and a *p*-≤0.01 were considered for further analysis.

### Isolation, siRNA transfection, and hormone treatment of human endometrial stromal cells

Endometrial stromal cells were isolated from tissue biopsies as previously described [80] with slight modifications. In brief, tissue samples were washed with DMEM-F12 (Life Technologies) containing 1% antibiotic-antimycotic and minced into small pieces of <1 mm^3^. The tissues were then incubated for 1.5 hours at 37°C in DMEM-F12 containing 0.25% (w/v) collagenase and 0.05% DNase I (Sigma-Aldrich). After enzymatic digestion, stromal cells were separated from epithelial aggregates using a 40 µm nylon cell strainer (BD-Biosciences). The filtrates were washed twice, and plated in DMEM-F12 media containing 10% fetal bovine serum and 1% antibiotic-antimycotic. Endometrial stromal cells were passaged two to three times before the experiments. Endometrial stromal cells were seeded in 6-well plates at a concentration of 1.5×10^5^ cells/ml, allowed to adhere and attain a confluency of approximately 60%. Cells were then transfected with non-targeting or Erbb siRNA (Thermo Scientific) using Lipofectamine 2000 or RNAiMAX (Life Technologies) according to manufacturer's protocol. All decidual experiments were conducted in opti-MEM media (Life Technologies) containing 2% charcoal-stripped fetal bovine serum and 1% antibiotic-antimycotic. Stromal cultures were treated with decidual hormones as described previously [49]. Decidualization was induced by treatment with 1 µM MPA (Sigma-Aldrich), 10 nM E2 (Sigma-Aldrich), and 50–100 µM dibutyryl cAMP (Sigma-Aldrich) every 48 hours.

### HESC immunofluorescence

HESC were grown on acid-etched glass coverslips (12-mm diameter) before transfection and decidualization. Subsequent to treatments, cells were washed once with PBS and fixed in 4% paraformaldehyde (Electron Microscopy Sciences) for 30 minutes. Excess fixative was quenched with freshly prepared sodium borohydride (NaBH4; 1 mg/ml). After quenching, cells were washed with TBS three times. Next, fixed cells were permeablized with 0.5% Triton X-100 for 30 minutes and washed with TBS three times. Non-specific antibody binding was blocked by 5% milk/TBS-T for 1 hour at room temperature. Primary antibodies (IGFBP1, 1∶200, Santa Cruz sc-6072; EGFR, 1∶200, Cell Signaling 2646) were diluted in blocking buffer and incubated overnight at 4°C. The following day, cells were washed with TBS and incubated with Alexa Fluor-conjugated secondary antibodies (Life Technologies, 1∶2000) in blocking buffer for 1 hour at room temperature. Then, cells were washed with TBS and post-fixed in 4% PFA for 10 minutes. Cells were washed and excess fixative quenched again in NaBH4 for 10 minutes. Finally, cells were treated with DAPI (1∶1000) for 20 minutes and HCS CellMask Blue (Life Technologies; 1∶10,000) for 1 hour at room temperature. Coverslips were rinsed quickly in water and mounted (SlowFade, Life Technologies) onto slides. Coverslips were imaged immediately.

### High throughput microscopy and image processing

Cells were imaged using the Cell Lab IC-100 Image Cytometer (IC-100; Beckman Coulter) equipped with a 40X/0.90NA objective. The imaging camera (Hamamatsu Orca ER) was set to capture 8 bit images at 2×2 binning with at least 4 images captured per field (DAPI, CellMask Blue, IGFBP1, EGFR). In general, >49 images were captured per coverslip. Images were analyzed using custom algorithms developed with the Pipeline Pilot (v8.0) software platform (Accelrys) in a similar workflow as previously described [81]–[83]. After background subtraction, nuclear and cell masks were generated using a combination of non-linear least squares and watershed-from-markers image manipulations of the DAPI images. Cell populations were filtered to discard events with cell aggregates, mitotic cells, apoptotic cells, cellular debris, or poor segmentation. Applied gates were based upon nuclear area, nuclear circularity, and cell size/nucleus ratio. All events with whole cell masks bordering the edge of the image were additionally eliminated from analysis. Post-analysis measurements were exported to spreadsheet software (Microsoft Excel) for further analysis.

### Assessment of kinase activity by Proteome Profiler Human Phospho-Kinase array kits

HESC were transfected with siRNA as mentioned above. Cells were split into two cohorts 24 hours after siRNA transfection. One cohort was serum-starved for 24 hours and then treated with decidual media (see above; containing 100 µM cAMP and 5% charcoal-stripped FBS). Treatment media was changed after 35.5 hours and cells were harvested 30 minutes later. The other cohort was double serum starved, starving for 12 hours overnight, refeeding with 10% serum for 12 hours, and then serum-starved again overnight. Cells were then treated with DMEM/F12 (Life Technologies) containing 100 ng/ml rhHB-EGF (R&D systems) for 30 minutes. Lysates were prepared using the supplied buffer. Proteome Profiler Human Phospho-Kinase array kits (R&D Systems) were used according to manufacturer's instructions to measure (in duplicate) changes to 43 different kinases and 2 related total proteins. A total of 500 µg of protein was used for each cohort. A total of 15 different x-ray exposures, ranging from 15 seconds to 15 minutes, were used to capture array signal. Signal intensity was quantified using ImageJ (NIH) and was normalized to the reference spots. Exposures with the greatest dynamic range for each pair of antibody spots were used to determine the fold change in signal intensity. Antibodies with an absolute intensity difference greater than 1.5 were chosen for representation.

### Statistical analysis

For comparisons involving two groups, unpaired two-tailed student's t-tests were performed. For comparisons involving three or more groups, a one-way ANOVA followed by Tukey's *post hoc* multiple-range test was conducted.

## Supporting Information

Figure S1 No overt defects in ovarian or oviduct function using the *Pgr^Cre^* to ablate *Egfr* expression. Conditional knockout mice (*d/d*) and their respective controls (*f/f*) were mated with wild-type males to investigate potential non-uterine defects. (**A**) 3-week old females were stimulated with gonadotropins and embryos were recovered from the oviduct at day 0.5 of pregnancy. (**B**) Blastocysts were flushed from the uterine lumen at day 3.5 of pregnancy from naturally cycling and mated females. (**C**) Serum progsterone concentration was measured at day 5.5 of pregnancy.
(TIF)

Figure S2 Normal decidualization in *Erbb2* and *Erbb3* conditional knockout mice. Conditional knockout mice (*d/d*) and their respective controls (*f/f*) were ovariectomized, administered exogenous hormones and one uterine horn was given a deciduogenic stimulus. (**A–D**) Images of gross uterine morphology of (**A**) *Erbb2^f/f^*, (**C**) *Erbb2^d/d^*, (**B**) *Erbb3^f/f^* and (**D**) *Erbb3^d/d^* mice 5 days after deciduogenic stimulus. Scale bars: 1 cm. (**E,F**) Wet weight measurements of stimulated uterine horns relative to the unstimulated horn of (**E**) *Erbb2* and (**F**) *Erbb3* females.
(TIF)

Table S1 A list of antigens recognized in the spotted human phospho-kinase antibody array (R&D Systems).
(TIF)

Table S2 A list of oligonucleotide sequences used for SYBR qPCR.
(TIF)
