# Supplementary figures and images for: The Epidermal Growth Factor Receptor Critically Regulates Endometrial Function during Early Pregnancy
Source: PLoS Genet. 2014 Jun 19;10(6):e1004451. doi: 10.1371/journal.pgen.1004451 (PMC4063709; doi:10.1371/journal.pgen.1004451)

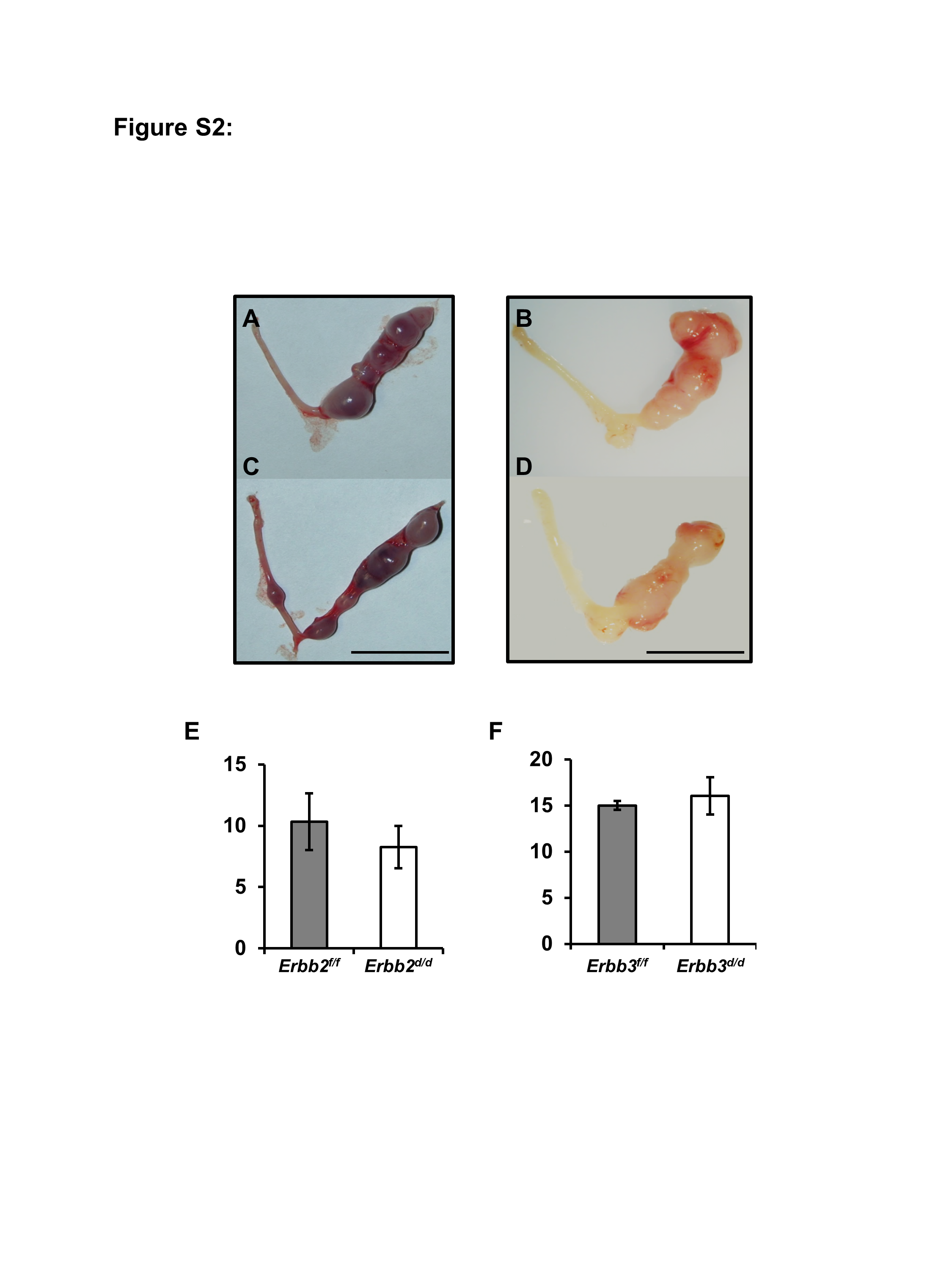

Supplement: Figure S2 — Normal decidualization in Erbb2 and Erbb3 conditional knockout mice. Conditional knockout mice (d/d) and their respective controls (f/f) were ovariectomized, administered exogenous hormones and one uterine horn was given a deciduogenic stimulus. (A–D) Images of gross uterine morphology of (A) Erbb2f/f, (C) Erbb2d/d, (B) Erbb3f/f and (D) Erbb3d/d mice 5 days after deciduogenic stimulus. Scale bars: 1 cm. (E,F) Wet weight measurements of stimulated uterine horns relative to the unstimulated horn of (E) Erbb2 and (F) Erbb3 females. (TIF) [file pgen.1004451.s002.tif]
